# Permanent draft genome sequence of sulfoquinovose-degrading *Pseudomonas putida* strain SQ1

**DOI:** 10.1186/s40793-015-0033-x

**Published:** 2015-07-22

**Authors:** Ann-Katrin Felux, Paolo Franchini, David Schleheck

**Affiliations:** Department of Biology, University of Konstanz, Konstanz, Germany; Konstanz Research School Chemical Biology, University of Konstanz, Konstanz, Germany; Genomics Center Konstanz, University of Konstanz, Konstanz, Germany

**Keywords:** *Pseudomonas putida* SQ1, aerobic, Gram-negative, *Pseudomonadaceae*, plant sulfolipid, organosulfonate, sulfoquinovose biodegradation

## Abstract

*Pseudomonas putida* SQ1 was isolated for its ability to utilize the plant sugar sulfoquinovose (6-deoxy-6-sulfoglucose) for growth, in order to define its SQ-degradation pathway and the enzymes and genes involved. Here we describe the features of the organism, together with its draft genome sequence and annotation. The draft genome comprises 5,328,888 bp and is predicted to encode 5,824 protein-coding genes; the overall G + C content is 61.58 %. The genome annotation is being used for identification of proteins that might be involved in SQ degradation by peptide fingerprinting-mass spectrometry.

## Introduction

*Pseudomonas putida* strain SQ1 belongs to the family of *Pseudomonadaceae* in the class of *Gammaproteobacteria*. The genus *Pseudomonas* was first described by Migula (in the year 1894 [1]) and the species *Pseudomonas putida* by Trevisan (in 1889 [2]). *P. putida* strain KT2440 was the first strain whose genome had been sequenced (in 2002 [3]), and it is the most well-studied *P. putida* strain thus far [4]. Currently, there are more than 30 genome sequences of *P. putida* strains available (e.g., 12 complete and 24 draft genomes in NCBI; January 2015), including the complete genome sequence of type strain NBRC 14164^T^ [5]. *P. putida* species are highly abundant in water, soil and in the rhizosphere [6, 7], can be plant-beneficial [8], and are extensively studied for their capabilities to degrade a broad range of substrates, especially aromatic compounds [9–12].

*P. putida* strain SQ1 was isolated for its ability to utilize the sulfonated plant sugar sulfoquinovose (6-deoxy-6-sulfoglucose) as a sole source of carbon and energy for growth, and was enriched from a sample of littoral sediment of pre-Alpine Lake Constance, Germany [13]. SQ is the polar headgroup of the plant sulfolipid sulfoquinovosyl diacylglycerol, which is present in the photosynthetic membranes of all higher plants, mosses, ferns and algae and most photosynthetic bacteria [14]. SQ is one of the most abundant organosulfur compounds in the biosphere, following glutathione, cysteine, and methionine, and the global production of SQ is estimated at 10 gigatons (10^10^ tons) per year [15]. Hence, the complete degradation of SQ concomitant with a recycling of the bound sulfur in form of inorganic sulfate is an important process of the carbon and sulfur cycle, e.g. in soils.

Until today only one bacterial degradation pathway for SQ has been identified, ‘sulfoglycolysis’ in *Escherichia coli* K-12 [16]. In this pathway, SQ is catabolized in analogy to glucose-6-phosphate *via* an adapted Embden-Meyerhof-Parnas (glycolysis) pathway, involving four newly identified enzymes and genes, and four newly identified metabolites. The pathway yields dihydroxyacetone phosphate (DHAP), which drives energy metabolism and growth of *E. coli*, and sulfolactaldehyde, which is reduced to dihydroxypropanesulfonate and excreted [16]. For *Pseudomonas* species, it is well-known that these bacteria lack the key enzyme for glycolysis, phosphofructokinase, but that the alternative Entner-Doudoroff pathway is operative, i.e., an oxidative entry into glucose-6-phosphate catabolism *via* a dehydrogenase enzyme. We detected a SQ-dehydrogenase activity in crude extract of SQ-grown *P. putida* SQ1 cells, and we therefore suspect that a ‘Sulfo-Entner-Doudoroff’-type of pathway might be operative in *P. putida* SQ1 for catabolism of SQ, but not sulfoglycolysis.

A draft genome sequence of strain SQ1 has been established and annotated in the IMG pipeline, and the annotation has been transferred to a proteomics (Mascot) database for peptide fingerprinting-mass spectrometry: in our present (unpublished) work, the database is used to identify enzymes and genes that are specifically induced during growth with SQ, e.g. in comparison to cells grown with glucose, by two-dimensional protein gel electrophoresis. Here, we present a summary classification and a set of features for *Pseudomonas putida* strain SQ1, together with the description of the shotgun genomic sequencing and annotation.

## Organism Information

### Classification and features

**Fig. 1:**
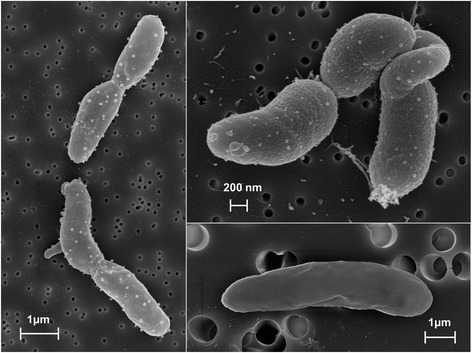
Scanning electron micrographs of *Pseudomonas putida* SQ1. Cells derived from a liquid culture (LB medium)

*P. putida* SQ1 is a rod-shaped (Fig. 1), motile, Gram-negative bacterium that grows aerobically in complex medium (e.g. LB-medium), or prototrophically in mineral-salts medium with a single carbon source (e.g., succinate, glucose, SQ). Strain SQ1 grows overnight on LB-agar plates and forms beige-whitish, smooth colonies (Table 1). *Pseudomonas putida* SQ1 has been deposited in the Leibniz Institute DSMZ-German Collection of Microorganisms and Cell Cultures under reference number DSM 100120.

**Fig. 2:**
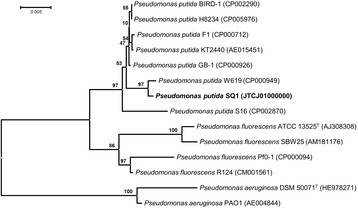
Phylogenetic tree based on the 16S rRNA gene sequence of *P. putida* SQ1, and sequences of other strains of the species *P. putida*, *P. aeruginosa* and *P. fluorescens*. The sequences were aligned with the CLUSTAL W program and the tree was built with the neighbor-joining algorithm integrated in the MEGA 6.0 program [31]. The phylogenetic tree was tested with 1000 bootstrap replicates; bootstrap values are shown at each node. The scale bar represents a 0.005 % nucleotide sequence divergence

Based on its 16S rRNA gene sequence, strain SQ1 is a member of the genus and species *Pseudomonas putida*, which is placed in the family *Pseudomonadaceae* within the order *Pseudomonadales* of *Gammaproteobacteria*, as illustrated by a phylogenetic tree shown in Fig. 2. Currently, 1,732 genome sequences of member of the order *Pseudomonadales* of *Gammaproteobacteria*, and 707 genome sequences within the family *Pseudomonadaceae* have been established (IMG JGI, January 2015).

## Genome sequencing information

### Genome project history

The DNA sample was submitted to GATC Biotech (Konstanz, Germany) in December 2012 where the whole-genome shotgun sequencing phase was completed in April 2013; the whole-genome shotgun sequencing was performed by GATC using the Illumina HiSeq2000 platform and a 100-bp paired-end library. After the mapping and *de-novo* assembly of the unmapped reads, which was done at the Genomics Center of the University of Konstanz, the draft genome sequence was uploaded into the IMG Pipeline for annotation and presented for public access on December 2014. The draft genome annotation is available at IMG under the IMG submission ID 14279, and was also deposited in Genbank under the accession number JTCJ00000000. Table 2 presents the project information and its association with MIGS version 2.0 compliance [17].

**Table 1:** Classification and general features of *Pseudomonas putida* SQ1 [32]

MIGS ID	Property	Term	Evidence code^a^
	Current classification	Domain *Bacteria*	TAS [33]
		Phylum *Proteobacteria*	TAS [34]
		Class *Gammaproteobacteria*	TAS [34, 35]
		Order *Pseudomonadales*	TAS [36, 37]
		Family *Pseudomonadaceae*	TAS [38, 39]
		Genus *Pseudomonas*	TAS [1, 38–40]
		Species *putida*	TAS [1, 2]
		Strain SQ1	TAS [13]
	Gram stain	Negative	TAS [13]
	Cell shape	Rod-shaped	TAS [13]
	Motility	Motile	TAS [13]
	Sporulation	Non-sporulating	TAS [13]
	Temperature range	Mesophile	TAS [13]
	Optimum temperature	30 °C	TAS [13]
	pH range; Optimum	Not tested; 7.2	TAS [13]
	Carbon source	Succinate, glucose, sulfoquinovose	IDA,TAS [13]
	Energy source	Chemoorganotroph	IDA,TAS [13]
MIGS-6	Habitat	Aerobic habitat	TAS [13]
MIGS-22	Oxygen requirement	Aerobic	TAS [13]
MIGS-15	Biotic relationship	Free-living	NAS
MIGS-14	Pathogenicity	Potentially pathogenic, Risk group 2 (classification according to German TRBA)	
MIGS-4	Geographic location	Isolated from littoral sediment of Lake Constance, Germany	TAS [13]
MIGS-5	Collection date	2011	TAS [13]
MIGS-4.1 MIGS-4.2	Latitude	47°41'44.77"N	
	Longitude	9°11'34.76"E	
MIGS-4.4	Altitude	399 m	

^a^Evidence codes – IDA: Inferred from Direct Assay; TAS: Traceable Author Statement (i.e., a direct report exists in the literature); NAS: Non-traceable Author Statement (i.e., not directly observed for the living, isolated sample, but based on a generally accepted property for the species, or anecdotal evidence). These evidence codes are from the Gene Ontology project [32]

**Table 2:** Project information

MIGS ID	Property	Term
MIGS-31	Finishing quality	Permanent draft
MIGS-28	Libraries used	100-bp paired-end library
MIGS-29	Sequencing platforms	Illumina HiSeq2000
MIGS-31.2	Fold coverage	>10x
MIGS-30	Assemblers	Velvet v1.2.10
MIGS-32	Gene calling method	Prodigal
	Genbank ID	JTCJ00000000
	Genbank Date of Release	December 16, 2014
	GOLD ID	Gi0045313
	BIOPROJECT	PRJNA266268
MIGS 13	Source Material Identifier	DSM 100120
	Project relevance	Study of unknown degradation pathway

### Growth conditions and genomic DNA preparation

Genomic DNA was extracted from an overnight culture of *P. putida* SQ1 grown at 30 °C in LB medium (500-ml scale), using JGI`s Bacterial Genomic DNA isolation protocol (CTAB protocol 2012).

### Genome sequencing and assembly

The whole-genome shotgun sequencing was performed under contract by GATC Biotech (Konstanz, Germany) using the Illumina HiSeq2000 platform and a 100-bp paired-end library, which resulted in 23,816,201 sequenced reads (1.85 × 10^9^ total bases). The trimming, mapping, as well as the *de novo* assembly of the unmapped raw reads, was performed at the Genomics Center of the University of Konstanz, Germany. First, the remaining adapters were removed and reads were trimmed by quality in CLC Genomics Workbench v6.5 (CLC bio, Aarhus, Denmark). In the next step, Bowtie v2.2.3 [18] was used to align the filtered reads against the genome of the closest relative, *P. putida* strain W619, to which 21,943,994 reads matched. These mapped reads were assembled with a reference-guided approach using the Columbus module implemented in Velvet v1.2.10 [19]. Velvet was then used to *de novo* assemble also all unmatched 1,872,207 reads (8.5 % of total reads). The whole process resulted in a total number of 1,634 contigs larger than 200 bp; the largest contig is 37,533 bp. The size of the draft genome is 5.3 Mb with 4,750,611 DNA coding bases, which is a normal size compared to other known *P. putida* genomes (range 3.0 to 7.1 Mb). The average G + C content is 61.58 %. At this time, no additional work is planned for this genome sequencing project (labeled as Permanent Draft).

### Genome annotation

Genes were identified and auto-annotated in the DOE-IMG pipeline [20]. Genes were identified using Prodigal [21] and the predicted CDGs were translated and used to search the National Center for Biotechnology Information (NCBI) nonredundant database, UniProt [22], TIGRFam [23], Pfam [24], KEGG [25], COG [26], and InterPro [27] databases. The tRNAscan-SE tool [28] was used to identify tRNA sequences, whereas ribosomal RNA sequences were identified by searches against models of the ribosomal RNA genes built from SILVA [29]. The RNA components of the protein secretion complex and the RNaseP were identified by searching the genome of the corresponding Rfam profiles using INFERNAL.

## Genome properties

The draft genome assembly of *P. putida* SQ1 consists of 1,634 contigs with an overall G + C content of 61.58 %. For these contigs, 5,925 complete genes or partial genes at ends of contigs have been predicted, 5,824 (98.30 %) of which for protein-coding genes. 4,624 (78.04 %) of these were assigned to a putative function with the remaining annotated as hypothetical proteins. The draft genome annotation predicted also 101 (1.70 %) sequences of RNA coding genes. The properties and the statistics of the draft genome annotation are summarized in Table 3 and the distribution of genes into COGs functional categories is presented in Table 4.

**Table 3:** Nucleotide and gene count levels of the genome of *P. putida* SQ1

Attribute	Genome (total)	
	Value	% of total^a^
Genome size (bp)	5,328,888	100.00
DNA coding	4,750,611	89.15
DNA G + C (bp)	3,281,384	61.58
DNA scaffolds	1,634	100.00
Total genes	5,925	100.00
Protein coding genes	5,824	98.30
RNA genes	101	1.70
rRNA operon count	9	0.15
Genes with function prediction	4,624	78.04
Genes in paralog clusters	4,497	75.90
Genes assigned to COGs	3,249	54.84
Genes with Pfam domains	4,781	80.69
Genes with signal peptides	535	9.03
Genes with transmembrane helices	1,270	21.43
CRISPR count	1	

^a^) The total is based on either the size of the genome in base pairs or the total number of protein coding genes predicted in the annotated draft genome

**Table 4:** Number of genes associated with general COG functional categories in *P. putida* SQ1

Code	Value	% age^a^	Description
J	167	4.60	Translation, ribosomal structure and biogenesis
A	1	0.03	RNA processing and modification
K	323	8.90	Transcription
L	107	2.95	Replication, recombinant and repair
B	1	0.03	Chromatin structure and dynamics
D	28	0.77	Cell cycle control, Cell division, chromosome partitioning
V	40	1.10	Defense mechanisms
T	204	5.62	Signal transduction mechanisms
M	179	4.93	Cell wall/membrane/envelope biogenesis
N	99	2.73	Cell motility
U	107	2.95	Intracellular trafficking, secretion, and vesicular transport
O	143	3.94	Posttranslational modification, protein turnover, chaperones
C	221	6.09	Energy production and conversion
G	183	5.04	Carbohydrate transport and metabolism
E	369	10.17	Amino acid transport and metabolism
F	87	2.40	Nucleotide transport and metabolism
H	158	4.35	Coenzyme transport and metabolism
I	147	4.05	Lipid transport and metabolism
P	216	5.95	Inorganic ion transport and metabolism
Q	91	2.51	Secondary metabolites biosynthesis, transport and catabolism
R	423	11.66	General function prediction only
S	335	9.23	Function unknown
-	2,676	45.16	Not in COGs

^a^) The total is based on the total number of protein coding genes in the annotated genome

Currently, there are 50 genome sequencing projects for *Pseudomonas putida* strains registered in the JGI Genomes Online Database (GOLD), and 32 *P. putida* genome sequences (finished or permanent draft) are accessible within the IMG database (January 2015) for direct comparison; their genome sizes range between 3.0 Mb (*P. putida* MR3) and 7.1 Mb (*P. putida* S12), and their overall G + C content ranges between 60.81 % (*P. putida* MR3) and 63.14 % (*P. putida* CSV86). The genome sequence of *P. putida* W619 was chosen as reference genome for the mapping, as this genome showed the highest overall nucleotide sequence identity (91.9 %) of all genomes of *P. putida* strains that had been available at the time of sequencing. For comparison, the genome of the most well-studied *P. putida* strain, strain KT2440, shows 49.3 % overall nucleotide sequence identity to that of strain SQ1.

The genome of strain SQ1 (5.3 Mb) is smaller compared to these of strains W619 (5.8 Mb) and KT2440 (6.2 Mb). The IMG abundance profiles for these three *P. putida* genomes indicated a lower abundance of transposases (COG3436 and COG3547) in strains SQ1 (2 total) and W619 (2 total) in comparison to KT2440 (21 total), as well as a lower abundance of ABC-type periplasmic, transmembrane or permease component genes (COG0834, COG0765, COG0715, COG0683, COG1132, COG0747 and COG4177) in strains SQ1 (46 total) and W619 (47 total) in comparison to KT2440 (68 total).

In the draft genome of *P. putida* SQ1, all genes for the Entner-Doudoroff pathway for glucose/glucose-6-phosphate are represented as part of the two gene clusters (operons) that are highly conserved within *P. putida* species (e.g., [30]), i.e., predicted genes for glucose-6-phosphate 1-dehydrogenase (IMG locus tag PpSQ1_03570), 6-phosphogluconolactonase (PpSQ1_03569) and 2-keto-3-deoxy-phosphogluconate aldolase (PpSQ1_03568) (gene cluster PP1022-24 in *P. putida* KT2440, respectively), and glucokinase (PpSQ1_04592), 6-phosphogluconate dehydratase (PpSQ1_02498/04591) and glyceraldehyde-3-phosphate dehydrogenase (gene cluster PP1011-09 in *P. putida* KT2440, respectively); notably, the prediction of the dehydratase gene is distributed over two contigs of the draft assembly (and therefore has two IMG locus tags), however, the respective contigs are contiguous, as confirmed by PCR with a primer pair spanning over both contigs (this study). Further, all genes for a periplasmic entry into the Entner-Doudoroff pathway (e.g., [30]) were predicted in the draft genome of *P. putida* SQ1, i.e., for membrane-bound PQQ-dependent glucose dehydrogenases (e.g., PpSQ1_02906) and gluconate dehydrogenase complex (e.g., PpSQ1_00542), and for gluconokinase (PpSQ1_05341), 2-ketogluconate kinase (PpSQ1_05601/ 02858) and 2-ketogluconate 6-phosphate reductase (PpSQ1_02860).

No candidate genes for a sulfoglycolytic pathway for SQ, as found in *E. coli* K12 [16], were detected in the draft genome sequence of strain SQ1, which supports the notion that a novel, alternative pathway for SQ is operative in strain SQ1 (see Introduction). Neither *P. putida* strains W619, KT2440 nor F1 grew with SQ when tested ([13] and this study). Further, our preliminary proteomic data (not shown) indicates that enzymes/genes of the ‘classical’ Entner-Doudoroff pathway for glucose/glucose-6-phosphate (see above) are highly induced during growth with glucose, as expected, but not during growth with SQ. We concluded that additional genes in *P. putida* strain SQ1 are involved in the utilization of SQ, and that these genes might be located on contigs that resulted from the *de novo* assembly of the un-mapped reads. If appropriate, the proteomic identification of the core enzymes of this novel SQ degradation pathway based on the draft genome sequence established in this study, and their confirmation by biochemical and analytical-chemical methods, will be reported in a future communication.

## Conclusions

Here, we present a summary classification and a set of features for *Pseudomonas putida* strain SQ1, together with the description of the shotgun genomic sequencing and annotation. The draft genome annotation contains no candidate genes for a sulfoglycolytic pathway for SQ, as found in *E. coli* K12, hence, the pathway operative in *P. putida* SQ1 represents a second, yet unknown bacterial degradation pathway for SQ. Furthermore, our preliminary proteomic data suggested that the ‘classical’ Entner-Doudoroff enzymes for a utilization of glucose/glucose-6-phophate are not induced during growth with SQ and that, hence, additional enzymes in strain SQ1 are operative during utilization of SQ. Based on the draft genome sequence, these enzymes and genes can now be defined.

## Abbreviations

SQ: sulfoquinovose
SQDG: sulfoquinovosyl diacylglycerol
DHAP: dihydroxyacetone phosphate
LB: lysogeny broth
ABC: ATP-binding cassette
